# Detection of Intrinsically Resistant Candida in Mixed Samples by MALDI TOF-MS and a Modified Naïve Bayesian Classifier

**DOI:** 10.3390/molecules26154470

**Published:** 2021-07-24

**Authors:** Jie Gong, Chong Shen, Meng Xiao, Huifang Zhang, Fei Zhao, Jiangzhong Zhang, Di Xiao

**Affiliations:** 1 State Key Laboratory of Infectious Disease Prevention and Control, Collaborative Innovation Center for Diagnosis and Treatment of Infectious Diseases, National Institute for Communicable Disease Control and Prevention, Chinese Center for Disease Control and Prevention, Changbai Road 155, Changping, Beijing 102206, China; gongjie@icdc.cn (J.G.); zhanghuifang@icdc.cn (H.Z.); zhaofei@icdc.cn (F.Z.); zhangjianzhong@icdc.cn (J.Z.); 2 Center for Statistical Science, Tsinghua University, Beijing 100084, China; c-shen18@mails.tsinghua.edu.cn; 3 Department of Industrial Engineering, Tsinghua University, Beijing 100084, China; 4 Department of Clinical Laboratory, Peking Union Medical College Hospital, Chinese Academy of Medical Sciences, Beijing 100032, China; cjtcxiaomeng@aliyun.com; 5 Beijing Key Laboratory for Mechanisms Research and Precision Diagnosis of Invasive Fungal Diseases, Beijing 100032, China

**Keywords:** MALDI TOF-MS, *Candida krusei*, *Candida auris*, coinfection, modified naïve Bayesian classifier

## Abstract

MALDI-TOF MS is one of the major methods for clinical fungal identification, but it is currently only suitable for pure cultures of isolated strains. However, multiple fungal coinfections might occur in clinical practice. Some fungi involved in coinfection, such as *Candida krusei* and *Candida auris*, are intrinsically resistant to certain drugs. Identifying intrinsically resistant fungi from coinfected mixed cultures is extremely important for clinical treatment because different treatment options would be pursued accordingly. In this study, we counted the peaks of various species generated by Bruker Daltonik MALDI Biotyper software and accordingly constructed a modified naïve Bayesian classifier to analyze the presence of *C. krusei* and *C. auris* in simulated mixed samples. When reasonable parameters were fixed, the modified naïve Bayesian classifier effectively identified *C. krusei* and *C. auris* in the mixed samples (sensitivity 93.52%, specificity 92.5%). Our method not only provides a viable solution for identifying the two highlighted intrinsically resistant *Candida* species but also provides a case for the use of MALDI-TOF MS for analyzing coinfections of other species.

## 1. Introduction

Matrix-assisted laser desorption/ionization time-of-flight mass spectrometry (MALDI-TOF MS) can be used for the rapid identification of fungal microorganisms by detecting specific ribosomal and other housekeeping proteins [1]. MALDI-TOF MS has become one of the standard methods for routine yeast identification in an increasing number of clinical laboratories due to its accuracy, cost, and speed [2,3]. However, it should be mentioned that the strains must be isolated and cultured before MALDI-TOF MS identification. To date, only a few studies have discussed the detection of mixed bacterial samples by MALDI-TOF MS [4,5]. However, no research has been conducted on the analysis of mixed samples involving fungi.

In fact, coinfection is a very common phenomenon in clinical practice. Coinfections involving fungi include viruses and fungi, bacteria and fungi, and different species of fungi (e.g., [6,7,8]). At present, the gold standard for the diagnosis of fungal infections is still culture [9]. For the culture of pathogenic fungi, Sabouraud dextrose agar (SDA) or potato dextrose agar (PDA) containing 0.1–0.5% chloramphenicol is mainly used. The culture conditions of viruses and bacteria are completely different from those of fungi. Therefore, bacteria and viruses generally cannot be detected with the culture of fungi. In contrast, if there are multiple fungal coinfections, the culture results are likely to show multiple fungi. Different fungi might have relatively large morphological differences. For example, some are filamentous fungi, and some are yeast-phase fungi. Therefore, we have focused our research on the identification of closely related species. More specifically, we are especially concerned with the coinfection of species within the *Candida* genus.

*Candida* is one of the most common pathogenic fungi that can cause invasive fungal disease [10]. Therefore, the identification of *Candida* sp. based on MALDI-TOF MS has always been a concern. It is currently believed that MALDI-TOF MS could be effectively used to identify *Candida* species [11,12]. As mentioned earlier, there is currently no research involving the use of MALDI-TOF MS to analyze mixed samples related to *Candida*. However, there have been reports of cases involving coinfections with multiple *Candida* species (e.g., [13]). Different *Candida* species may have completely different drug-resistance characteristics. As a result, different treatment options need to be used in clinical practice. In particular, some *Candida* species are intrinsically resistant to specific antifungal drugs [14]. For example, *Candida krusei* is intrinsically resistant to fluconazole [15,16]. Recently, the newly emerged *Candida auris* has become another famous intrinsically resistant *Candida* [17]. Additionally, there have been cases of *C. krusei* coinfecting with other *Candida* species [18].

Therefore, we constructed a new set of statistical methods to analyze whether isolated *Candida* strains include naturally resistant strains. MALDI TOF-MS analysis of mixed fungal samples has not been reported previously. Therefore, the significance of this study is not only to provide a method for effectively identifying two important intrinsically drug-resistant *Candida* species from coinfected samples but also to provide a new strategy for analyzing the problem of fungal coinfection.

## 2. Methods and Materials

### 2.1. Strains and Simulated Samples

There were 62 fungal isolates used in this study. These strains were collected from the national surveillance program for invasive fungal infections (the CHIF-NET study, Table 1, Supplement A). The isolates were stored at −80°C until use at Peking Union Medical College Hospital, Beijing, China (PUMCH). The strains were identified by morphological analysis with CHROMagar^TM^ Candida medium (Difco Laboratories, Detroit, MI, USA) and sequencing of the rDNA internal transcribed spacer (ITS) and secondary alcohol dehydrogenase (SADH) [19,20].

The simulated samples were composed of pure-cultured fungi mixed in defined proportions (Table 1, Supplement A and B). The cultured fungi were suspended in sterile saline solution and measured by Densicheck (Biomerieux, l’Etoile, Marcy l’Etoile, France). The suspensions were adjusted to a McFarland 0.5 turbidity standard and mixed in defined proportions.

This study was approved by the ethics committee of the National Institute for Communicable Disease Control and Prevention (ICDC) and is consistent with the guidelines of the Declaration of Helsinki.

### 2.2. MALDI-TOF MS Sample Preparation

The supernatant of the mixed strains was removed after centrifugation (12,000× *g*) for 10 min. The precipitate was extracted by the ethanol/formic acid method, and the specific procedures were as follows. The precipitate was suspended in 300 μL of molecular-grade water and vortexed, and 900 μL of anhydrous ethanol was added. The samples were vortexed and centrifuged (13,000× *g*) for 2 min. The supernatant was discarded, and 50 μL of 70% formic acid was added and mixed. Finally, 50 μL of acetonitrile was added, and the solution was carefully mixed. After centrifugation (13,000× *g*) for 2 min, the supernatant was retained as the prepared sample. Then, 1 μL of supernatant was dropped on the sample target (MSP 96 ground steel 600-µm sample target). Three spots were prepared for each sample. After the sample dried naturally, it was covered with 1 μL of α-cyano-4-hydroxycinnamic acid (CHCA) (saturated matrix solution in 50% acetonitrile and 2.5% trifluoroacetic acid) and further dried naturally.

### 2.3. MALDI-TOF MS Data Acquisition and Processing

A Microflex LRF (Bruker Daltonics, Billerica, MA, USA) mass spectrometer was used for data acquisition. The software program used for the data acquisition was FlexControl (version 3.4, Bruker Daltonics). The parameters used were as follows: N2 laser (λ = 377 nm); mass range, 2000–20,000 Da; ion source 1, 20 kV; ion source 2, 18.5 kV; lens, 8.45 kV; pulsed ion extraction, 320 ns; and laser frequency, 20.0 Hz. Each spectrum was obtained by using 100 shots, and the spectra obtained after 500 shots were superimposed to generate the total spectrum. The *Escherichia coli* standard kit developed by the China CDC was used for mass calibration and instrument parameter optimization. One spectrum was acquired for each sample spot (spectra were acquired for each sample). Flex Analysis (version 3.4, Bruker Daltonics) was used to evaluate the quality of the spectra.

### 2.4. Statistical Analysis and Construction of a Modified Naïve Bayesian Classifier

Using MALDI Biotyper Compass Explorer (version 4.1) (Bruker Daltonics) software, the characteristic peaks of 269 Candida strains from 58 species were exported from the commercial database for species identification. These peaks were used as training data sets (Supplement C). There were 70 characteristic peaks of each strain, and the mass range was between 2000–20,000 Da.

We proposed a modified naïve Bayesian classifier to determine whether samples contained *C. krusei* or *C. auris* as well as the specific classification. The modified naïve Bayesian classifier first determined whether a sample contained *C. krusei* or *C. auris* based on the number of matched characteristic peaks and then determined the classification according to the posterior probability.

We compared the mass spectrometry data from different samples of the same strain and different strains. We found that there were characteristic peaks in different strains; that is, these peaks appeared frequently in different samples of the strain. For example, from 3240 to 3250, mass spectrometry data were present in all *C. krusei* samples but none of the *C. auris* samples; from 10,140 to 10,150, mass spectrometry data were present in all *C. auris* samples but none of the *C. krusei* samples. We selected these characteristic peaks as attributes of the Bayesian model.

In the Bayesian model, we calculated the posterior probability of all classes based on the input attribute $x=\left\{x^{1},\ldots ,x^{n}\right\}$ and took the class with the highest posterior probability as the output according to Formula (1) as follows:

(1) $$\hat{y}=argmax_{c_{k}}P\left(Y=c_{k}\right)\overset{n\left(m\right)}{\underset{j=n\left(1\right)}{\prod }}P(X^{j}=x^{j}\;|Y=c_{k})$$

where {n(1),…,n(m)}∈{1,2,…,n} is the index of the matched characteristic peaks and $c_{k}\in \left\{C.\;krusei,\;C.\;auris\right\}$ is the class. We assumed that the prior distribution $P\left(Y=c_{k}\right)$ was uniformly distributed. Since some attributes may not appear in the sample, we introduced Laplacian smoothing to calculate the conditional probability according to Formula (2) as follows:

(2) $$P(X^{j}=x^{j}\;|Y=c_{k})=\frac{m_{kj}+\lambda }{m_{k}+O_{j}\lambda }$$

where $m_{k}$ is the total number of $c_{k}$ samples, and $m_{kj}$ is the number of times that the jth attribute appears in the $c_{k}$ samples. λ is a constant greater than 0, and we always took λ=1. $O_{j}$ as the number of values for the jth attribute.

We set a threshold $m_{\alpha }$ to determine whether the sample contained the target species. We considered the sample to contain *C. krusei* or *C. auris* only when the number of matched characteristic peaks $m\geq m_{\alpha }$ and then judged whether the sample contained *C. krusei* or *C. auris* based on the posterior probability.

ROC curve, AUCs and Cohen’s kappa statistics were used to assess the performance of our classification model. All analyses were performed using R version3.5.1 (R Core Team, Vienna, Austria) [21,22].

## 3. Results

### 3.1. Modified Naïve Bayesian Classifier

We used the training data from Supplement C to determine characteristic peaks and corresponding conditional probabilities. Specifically, the mass spectrometry data ranging from 3000 to 14,150 were divided into intervals of 10. An interval containing 6 or more (17 in total) *C. krusei* samples and an interval containing 4 or more (9 in total) *C. auris* samples were selected. These peaks were combined as characteristic peaks, i.e., attributes of the Bayesian model (Supplement D). The corresponding conditional probability was calculated according to Formula (2). The modified naïve Bayesian classifier was constructed in R language (source code in Supplement E).

**Figure 1:**
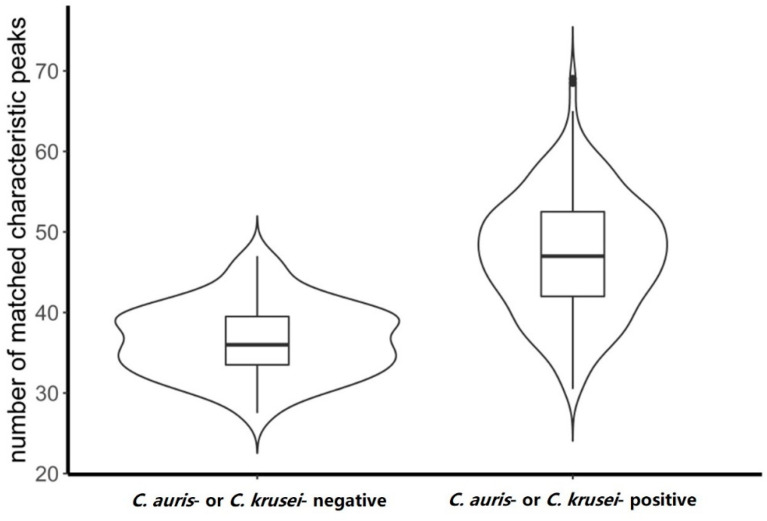
Violin plot of the number of matched characteristic peaks *C. auris*- or *C. krusei*-positive and -negative samples. Wider sections in the violin plots represent a higher probability; the skinnier sections represents a lower probability. The boxplot inside shows that the lower end of the box represents the first quartile while the upper end was the third quartile; the median value is marked with the bold black line in the center of the box.

We compared the number of matched characteristic peaks between *C. auris*- or *C. krusei*-positive and -negative samples. From Figure 1, we found that the negative sample had a bimodal distribution, with two peaks at 34 and 39. The positive samples showed a unimodal distribution with a peak value of 49. The variance of the positive sample was relatively large, which was related to the proportion of the target species. The matched characteristic peak in the positive samples was significantly higher than that in the negative samples (*p*-value < 2.2 × 10^−16^). Furthermore, the median of the positive samples was 47, the lower quartile was 42, and the upper quartile was 52; the median of the negative samples was 36, the lower quartile was 33, and the upper quartile was 39. There was a clear difference in m between the two types of samples. Specifically, m in the included samples was significantly higher than that in the nonincluded samples. Therefore, we used m to determine whether the samples contained the target species.

**Figure 2:**
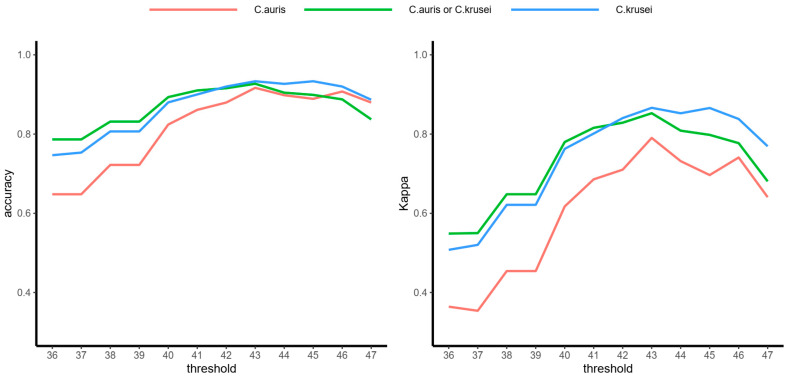
Accuracy and kappa values for *C. auris* and *C. krusei* mixed samples. Accuracy is the percentage of correctly classified samples out of all samples. Cohen’s kappa is the normalized accuracy at the baseline of a random guess. It is more useful when dealing with imbalanced data. The closer these two values were to 1.0, the better the model’s performance.

We then chose the threshold $m_{\alpha }$ based on the accuracy and kappa values of the samples. With the increase in $m_{\alpha }$, the accuracy and kappa values of *C. auris* or *C. krusei* mixed samples showed a trend of first increasing and then decreasing (Figure 2). When we took $m_{\alpha }=43$, both values reached the maximum, that is, accuracy=0.927 and kappa=0.853. In this case, the expected accuracy of the model was 0.476. This result indicated that the model had a perfect performance in predicting whether the mixed sample contained *C. auris* or *C. krusei.* Therefore, we took $m_{\alpha }=43$, and when the number of matched characteristic peaks was greater than 43, the sample was considered to contain the target species; otherwise, it was not.

### 3.2. Identification Results from the Modified Naïve Bayesian Classifier and Bruker Biotyper

The identification results from the modified naïve Bayesian classifier and Bruker Biotyper are shown in Table 2 and Supplement B.

In this study, 128 samples mixed at a 1:1 ratio were used (Supplement B). Ninety-eight of 128 samples contained *C. auris* or *C. krusei*. With the Bruker Biotyper, only 10 out of 98 samples were identified (Table 2). Among them, none of the samples containing *C. auris* were identified. In contrast, the modified naïve Bayesian classifier effectively identified 91 out of the 98 samples (92.86). Specifically, 89.28% of *C. auris* (25/28) samples and 94.29% of *C. krusei* (66/70) samples were identified separately. When the target species and nontarget species were mixed 1:10, the Bruker Biotyper did not identify any of the target species from the 92 samples (Table 2, Supplement B). In contrast, the modified naïve Bayesian classifier identified 23.66% of the samples as having these species (22/92).

**Figure 3:**
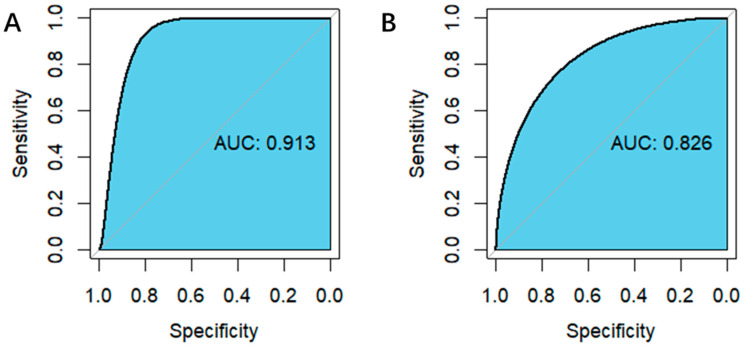
Receiver operating characteristic (ROC) curve and the area under the ROC curve (AUC). Specificity represents the proportion of the negative samples that were correctly classified, and sensitivity is the proportion of the positive samples that were correctly classified. AUC value is equivalent to the probability that a randomly chosen positive example is ranked higher than a randomly chosen negative example. The closer the ROC curve reaches the top left corner and the closer the AUC value is to 1, the better the classifier is. (**A**) 1:1 mixed sample. (**B**) 1:10 mixed sample.

The receiver operating characteristic (ROC) curve showed the details of the classifier on the 1:1 and 1:10 mixed samples (Figure 3). The areas under the ROC (AUCs) were 0.913 and 0.826, respectively. This demonstrated that our model performed well in predicting whether the mixed sample contained *C. auris* or *C. krusei*, especially in samples mixed at a 1:1 ratio. In other words, our model could not only identify samples that were *C. auris* or *C. krusei* positive, but also distinguish which sample was included.

To test the specificity of the methods, we also used 80 samples that did not contain the target species (*C. auris* or *C. krusei*). Among them, there were 50 samples of pure cultured strains and 30 samples of 1:1 mixtures of two species. For the samples of pure cultured strains, the Bruker Biotyper correctly identified 98% of the samples (49/50). For the 30 mixed samples, although the Bruker Biotyper did not mistakenly identify samples as containing the target species, it did not effectively identify the species in the samples. Correspondingly, the modified naïve Bayesian classifier excluded 94% of the pure culture strains and 90% of the mixed samples.

When the ratio of target species to nontarget species in the mixed sample was 1:10, it was difficult to identify the target species (Table 2, Supplement B). No sample containing the target species was effectively identified with the Bruker Biotyper. Correspondingly, the modified naïve Bayesian classifier recognized only 7.14% of *C. auris* samples (2/28) and 30.77% of *C. krusei* samples (20/64).

## 4. Discussion

In clinical and agricultural applications, the drug resistance of fungi has always been a concern [23]. The intrinsic resistance of fungi is also a very important issue in fungal drug resistance. *C. krusei* and *C. auris*, which were the target species of this study, are both intrinsically resistant pathogenic fungi. *C. krusei* is one of five most common *Candida* pathogens, accounting for approximately 1.4–2% of invasive candidiasis cases [10,21]. *C. auris* is an emerging pathogenic fungus that has received increasing attention in recent years. Therefore, we selected these two species to explore the potential of MALDI-TOF MS in the diagnosis of fungal coinfection.

In this study, we tried to simulate various conditions of coinfection involving *C. krusei* and *C. auris*. A newly established modified naïve Bayesian classifier and traditional Bruker Biotyper method were used to analyze raw data. The Bruker Biotyper effectively identified 1:1 mixed samples at the genus level (90/98, 91.83%), but it was difficult to effectively identify samples at the species level using this method (10/98, 10.2%) (Supplement B). This may be because too many tanglesome characteristic peaks interfered with the software’s effective judgment. However, the modified naïve Bayesian classifier effectively identified these samples. The naïve Bayesian classifier showed good robustness and was not sensitive to missing data. The MALDI-TOF MS data satisfied the assumption that the attributes were independent of each other. Comparing data from different species, we found that there were characteristic peaks common to the same species. However, the naïve Bayesian classifier could determine only whether a sample belonged to a certain type of fungus but not whether it contained a certain type of fungus. Therefore, for mixed samples, we first used the threshold $m_{\alpha }$ to determine whether the sample contained the target species and then used the posterior probability to determine which specific species were included. Changes in the threshold $m_{\alpha }$ affected the accuracy and kappa values.

It was noted that the samples mixed with *C. krusei* were more accurately identified than those mixed with *C. auris* whether the modified naïve Bayesian classifier (94.29% vs. 89.28%) or Bruker Biotyper (14.28% vs. 0%) method was used. This phenomenon may be related to the characteristics of *C. krusei* and *C. auris* because *C. krusei* has a more independent evolutionary status in the phylogenetic tree than *C. auris* [24]. More specifically, this independent evolutionary status may mean more unique biological characteristics, so *C. krusei* may be more easily identified.

When the target species and nontarget species were mixed 1:10, neither the modified naïve Bayesian classifier nor the Bruker Biotyper effectively identified the target species. This may be because the characteristic peaks of the target species were obscured by those of the nontarget species. The modified naïve Bayesian classifier effectively identified some samples (22/92, 23.91%) but still did not meet the requirements of clinical use. According to the samples that were identified by the modified naïve Bayesian classifier, certain species such as *Candida orthopsilosis* were more easily identified when they were interfered with (Supplement B). This might imply that, when some species are used as interferences, the target species would be more easily identified. In other words, with the same mixing ratio, some species can be identified as interferences, but some cannot. Therefore, it could be speculated that the detection limit of the modified naïve Bayesian classifier will change with the change in target species and nontarget species.

Clinical specimens, such as blood samples, cannot usually be directly used for MALDI TOF-MS analysis. First, too many interference impurities in clinical specimens might affect MALDI TOF-MS analysis. Second, there were too few pathogens in clinical specimens, which might not reach the lower limit of detection of MALDI TOF-MS analysis. In most cases, only cultured fungal strains would be used for MALDI TOF-MS analysis. Therefore, the focus of this study was on the strains obtained from culture, rather than directly on clinical specimens. In this study, we constructed a new algorithm, and hope to bring inspiration to other similar works, such as the analysis of bacterial or filamentous fungal mixed infections.

## Figures and Tables

**Table 1:** Candida strains and simulated samples used in this study.

Samples	Proportions	Numbers
*Candida albicans*	-	10
*Candida tropicalis*	-	10
*Candida haemulonis*	-	5
*Candida parapsilosis*	-	10
*Candida orthopsilosis*	-	5
*Candida metapsilosis*	-	5
*Candida glabrata*	-	5
*Candida auris*:*Candida albicans*	1:1	4
*Candida auris*:*Candida albicans*	1:10	4
*Candida auris*:*Candida tropicalis*	1:1	4
*Candida auris*:*Candida tropicalis*	1:10	4
*Candida auris*:*Candida glabrata*	1:1	4
*Candida auris*:*Candida glabrata*	1:10	4
*Candida auris*:*Candida parapsilosis*	1:1	4
*Candida auris*:*Candida parapsilosis*	1:10	4
*Candida auris*:*Candida orthopsilosis*	1:1	4
*Candida auris*:*Candida orthopsilosis*	1:10	4
*Candida auris*:*Candida metapsilosis*	1:1	4
*Candida auris*:*Candida metapsilosis*	1:10	4
*Candida auris*:*Candida haemulonis*	1:1	4
*Candida auris*:*Candida haemulonis*	1:10	4
*Candida krusei*:*Candida albicans*	1:1	10
*Candida krusei*:*Candida albicans*	1:10	10
*Candida krusei*:*Candida tropicalis*	1:1	10
*Candida krusei*:*Candida tropicalis*	1:10	8
*Candida krusei*:*Candida glabrata*	1:1	10
*Candida krusei*:*Candida glabrata*	1:10	10
*Candida krusei*:*Candida parapsilosis*	1:1	10
*Candida krusei*:*Candida parapsilosis*	1:10	10
*Candida krusei*:*Candida orthopsilosis*	1:1	10
*Candida krusei*:*Candida orthopsilosis*	1:10	9
*Candida krusei*:*Candida metapsilosis*	1:1	10
*Candida krusei*:*Candida metapsilosis*	1:10	10
*Candida krusei*:*Candida haemulonis*	1:1	10
*Candida krusei*:*Candida haemulonis*	1:10	8
*Candida albicans*:*Candida tropicalis*	1:1	2
*Candida albicans*:*Candida haemulonis*	1:1	2
*Candida albicans*:*Candida parapsilosis*	1:1	2
*Candida albicans*:*Candida orthopsilosis*	1:1	2
*Candida albicans*:*Candida glabrata*	1:1	2
*Candida tropicalis*:*Candida haemulonis*	1:1	2
*Candida tropicalis*:*Candida parapsilosis*	1:1	2
*Candida tropicalis*:*Candida orthopsilosis*	1:1	2
*Candida tropicalis*:*Candida glabrata*	1:1	2
*Candida haemulonis*:*Candida parapsilosis*	1:1	2
*Candida haemulonis*:*Candida orthopsilosis*	1:1	2
*Candida haemulonis*:*Candida glabrata*	1:1	2
*Candida parapsilosis*:*Candida orthopsilosis*	1:1	2
*Candida parapsilosis*:*Candida glabrata*	1:1	2
*Candida orthopsilosis*:*Candida glabrata*	1:1	2

**Table 2:** Identification results obtained with the modified naïve Bayesian classifier and Bruker Biotyper.

Sample Types	Intrinsically Resistant Candida	Accuracy-Threshold 43–Naïve Bayesian Classifier	Accuracy-Bruker Biotyper
1:1 mixed sample	*Candida auris*	25/28, 89.28%	0/28, 0%
1:1 mixed sample	*Candida krusei*	66/70, 94.29%	10/70, 14.28%
1:1 mixed sample	*Candida auris* or *Candida krusei*	91/98, 92.86%	10/98, 10.20%
1:10 mixed sample	*Candida auris*	2/28, 7.14%	0/28, 0%
1:10 mixed sample	*Candida krusei*	20/64, 30.77%	0/64, 0%
1:10 mixed sample	*Candida auris* or *Candida krusei*	22/92, 23.66%	0/92, 0%
Candida sp. Strains *	None	47/50, 94.00%	49/50, 98%
1:1 mixed sample *	None	27/30, 90.00%	-

*, The target species (*Candida auris* and *Candida krusei*) were not included in the sample.

## Data Availability

Not applicable.

## Supplementary Materials

The following are available online. Supplement A: strains used in this study, Supplement B: Identification results of modified naïve Bayesian classifier with different thresholds, Supplement C: characteristic peaks exported from the commercial database for species identification, Supplement D: characteristic peaks of *C. krusei* and *C. auris,* Supplement E: source code of modified naïve Bayesian classifier.

## Sample Availability

Samples of the compounds are available from the authors.
